# The CiWOX13-CiWOX14 Complex Regulates *CiBGLU21* to Promote Graft Union Formation by Modulating Cell Wall Synthesis in *Carya illinoinensis*

**DOI:** 10.3390/plants15020273

**Published:** 2026-01-16

**Authors:** Piyu Ji, Wanchun Li, Liangye Huang, Qinyuan Shen, Ying Yang, Ying Yang, Gaotian Chen, Muhammad Junaid Rao, Anket Sharma, Jianfang Zuo, Vijay Pratap Singh, Huwei Yuan, Bingsong Zheng

**Affiliations:** 1 State Key Laboratory for Development and Utilization of Forest Food Resources, Zhejiang Agriculture and Forestry University, Hangzhou 311300, China; jipiyu123@163.com (P.J.); liwanchun@stu.zafu.edu.cn (W.L.); 2023602121036@stu.zafu.edu.cn (L.H.); 2024202011016@stu.zafu.edu.cn (Q.S.); sky_yangying@163.com (Y.Y.); yingy@zafu.edu.cn (Y.Y.); chengaotian@stu.zafu.edu.cn (G.C.); mjr@zafu.edu.cn (M.J.R.); anketsharma@gmail.com (A.S.); zuojianfang@zafu.edu.cn (J.Z.); vijaypratap.au@gmail.com (V.P.S.); 2 Zhejiang Key Laboratory of Non-Wood Forest Products Quality Regulation and Processing Utilization, Zhejiang A&F University, Hangzhou 311300, China; 3 College of Modern Agricultural Sciences, Zhejiang Wanli University, Ningbo 315200, China; 4 Institute of Genomics for Crop Abiotic Stress Tolerance, Department of Plant and Soil Science, Texas Tech University, Lubbock, TX 79409, USA; 5 Plant Physiology Laboratory, Department of Botany, C.M.P. Degree College, A Constituent Post Graduate College of University of Allahabad, Prayagraj 211002, India

**Keywords:** *Carya illinoinensis*, β-glucosidase (BGLU), graft union formation, cell wall biosynthesis, WOX transcription factors

## Abstract

Grafting is an important method for pecans, while the molecular mechanisms underlying graft union formation still need in-depth analysis. In the current investigation, we identified 22 *BGLU* genes in *Carya illinoinensis* (pecan) and demonstrated that *CiBGLU21*, a β-glucosidase-encoding gene, plays an important positive role in graft healing. The overexpression of *CiBGLU21* enhanced graft survival rates and accelerated tissue regeneration, while biochemical assays confirmed its role in cell wall reinforcement and sugar metabolism. Additionally, we identified that CiWOX13 formed heterodimers with CiWOX14 to directly and synergistically activate the transcription of *CiBGLU21.* The current investigation revealed a CiWOX13/14*-CiBGLU21* module as an important modulator of graft union formation, offering insights into improving grafting efficiency in perennial crops and advancing the understanding of cell wall dynamics during tissue regeneration.

## 1. Introduction

Grafting technology has a long history and has played a pivotal role in the development of the plant industry. Many economically important tree species, as well as annual field crops, are propagated through grafting to combine desirable traits from both rootstocks and scions. Grafting serves as an effective strategy for enhancing agronomic performance, including an improved resistance to biotic and abiotic stresses [1], increased yield [2], enhanced nutrient utilization efficiency, and the induction of plant dwarfing [3]. Although grafting techniques may vary significantly across species, the formation of connective tissue between the rootstock and scion remains fundamental to graft success [4]. The graft healing process comprises four primary developmental stages, which include necrotic layer formation, callus proliferation, the differentiation of a new vascular cambium, and vascular bundle reconnection [2]. This complex biological process involves coordinated signal transduction and substantial material accumulation. Genes associated with wound responses [5,6,7], cell wall modification [8,9], secondary metabolism [10,11], and hormone signaling pathways have been shown to exhibit differential expression during grafting [1,12]. Cell wall modification and reconstruction are critical for successful graft formation [13,14]; the major components of plant secondary cell walls include cellulose, hemicellulose, and lignin [15]. Notably, genes involved in lignin and cellulose biosynthesis have been upregulated during graft healing in litchi [16], pecans [17], and pear [18].

In the graft healing process, a rapid transcriptional response occurs, including the upregulation of multiple genes related to cell wall biogenesis. The WUSCHEL-RELATED HOMEOBOX (WOX) family genes play a pivotal role in regulating stem cell formation and maintenance across diverse plant species, with WUSCHEL (WUS) specifically maintaining stem cell population homeostasis within the shoot apical meristem. During graft union formation, WOXs are the key regulators that control callus formation [19], organ reconnection, and vascular reconnection (Kim et al. 2018) [20]. WOX13 is rapidly induced upon wounding and directly activates genes encoding cell-wall-modifying enzymes, including *EXPANSINs* (EXPs), *PECTATE LYASE LIKE*s (PLLs), and *GH9B3* [9]. At the same time, WOX4 and WOX14 promote cambial cell proliferation, helping to form continuous xylem connections across graft junctions and facilitating vascular regeneration [2,21,22].

β-glucosidases (BGLUs) are members of the glycoside hydrolase 1 (GH1) family found in all domains of living organisms, with crucial roles in various biological processes [23]. In recent years, a growing number of β-glucosidase family members have been identified, leading to a better understanding of the genes that encode these enzymes. In plants, BGLU was clarified to be associated with diverse biological functions, including cell wall remodeling [24], forming lignin precursors [25,26], plant secondary metabolites [27], activating plant hormones, and responding to stresses [28,29]. β-glucosidases play essential roles in cell wall metabolism across a wide range of plant species. For instance, Os4BGLU12 hydrolyzes cell wall β-glucan-derived oligosaccharides during development or wounding [30]. In *Arabidopsis thaliana*, the stem-specific enzymes *AtBGLU45* and *AtBGLU46* are critical for lignin biosynthesis [25]. Similarly, in rice *Os4BGlu14*, *Os4BGlu16*, and *Os4BGlu18*, members of the β-glucosidase family, function directly in lignin synthesis [26], while *PtrBGLU6* is essential for lignification and cell wall formation in poplars [24]. In addition, a *BGLU* gene in pears, *PbBGLU13*, displayed a different expression level in the graft healing process, which probably affected the graft junction by encoding β-glucosidase [18].

*Carya illinoinensis* (pecan), a member of the Juglandaceae family, is an economically important nut crop cultivated in several countries, including China, owing to its nutritional, medicinal, and industrial value [17,31]. It is also widely used as a rootstock to improve the agronomic traits of *Carya cathayensis* (Chinese hickory) through grafting in Zhejiang and Anhui Provinces, China [32]. Although previous studies have reported key insights into the grafting process in pecans, the specific molecular mechanisms underlying the function of the BGLU gene family remain largely uncharacterized [10,17].

A thorough analysis of the *BGLU* gene family in *C. illinoinensis* and a function analysis represent significant implications for the development and utilization of the pecan industry. In the current study we investigated the *CiBGLU* gene family, identifying *CiBGLU21* as a crucial gene for *C. illinoinensis* graft union formation by increasing the activities of β-glucosidase, secondary metabolites of cell wall formation, and sugar accumulation. Additionally, we found that CiWOX13 and CiWOX14 interact with each other and co-promote the expression of *CiBGLU21*. The findings of the current investigation lay the foundation for future explorations of *BGLU* genes’ function and their roles in the regulation of graft unions in *C. illinoinensis* and potentially other economically important plants.

## 2. Results

### 2.1. CiBGLU21 Plays an Important Positive Role in Graft Union Formation

**Figure 1:**
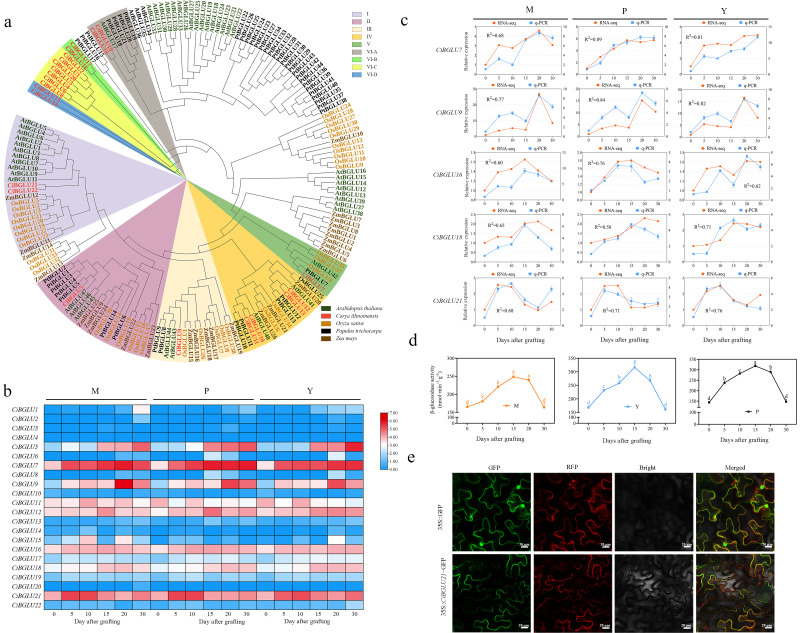
Phylogenetic and expression analysis of *CiBGLU* genes and activity of β-glucosidase of different grafting combinations at 0, 5, 10, 15, 20, and 30 days after grafting. (**a**) Phylogenetic analysis of BGLUs from *C. illinoinensis*, *A. thaliana*, *Oryza sativa*, *Zea mays,* and *Populus trichocarpa*. (**b**) Heat map of expression patterns for *CiBGLU* genes. (**c**) Relative expression of *CiBGLU* genes. (**d**) Activity of β-glucosidase of different grafting combinations. M, the rootstock of grafting combination is Mahan; P, the rootstock of grafting combination is Pawnee; and Y, the rootstock of grafting combination is Yalin. (**e**) Subcellular localization of CiBGLU21. Values represent mean ± SD; different letters represent a significant difference via Duncan’s multiple range test.

A total of 22 *BGLU* genes were identified by using the local BLAST program v2.15.0 and the Pfam tool to search the whole genome of *C.illinoinensis*, named *CiBGLU1*-*CiBGLU22*. In addition, we analyzed the phylogenetic, gene structure, collinearity, chromosomal localization, and cis-acting regulatory elements of *CiBGLU* genes (Figure 1a, Supplementary Figures S1 and S2). To explore the expression levels of *CiBGLU* family genes during the graft healing process of three grafting combinations with different C. illinoinensis rootstocks, transcriptome sequencing was performed on the grafting junction at 0, 5, 10, 15, 20, and 30 days after grafting. During the healing process, a high expression was observed in nine *CiBGLU* genes, including *CiBGLU5*, *CiBGLU7*, *CiBGLU9*, *CiBGLU11*, *CiBGLU12*, *CiBGLU16*, *CiBGLU17*, *CiBGLU18*, and *CiBGLU21*, while expression levels of other genes were significantly lower (Figure 1b). Based on the expression levels of *CiBGLU* genes, primers of five *CiBGLU* genes, including *CiBGLU7*, *CiBGLU9*, *CiBGLU16*, *CiBGLU18*, and *CiBGLU21,* were selected and designed to conduct a qRT-PCR analysis for expression validation, and the findings were consistent with the transcriptomic analysis results (Figure 1c). The activity of the β-glucosidase of different grafting combinations was tested by analyzing the expression pattern of the *CiBGLU21* expression, suggesting that its enzymatic activity is critical for graft union formation (Figure 1d).

In order to verify the subcellular localization of CiBGLU21 predicted by the Psort-II server, we constructed each CiBGLU21-GFP vector and analyzed the transient expression in tobacco leaves. The results indicated that the fluorescent signals of the CiBGLU21-GFP fusion vectors were coincident with the signal of the cell membrane marker protein (Figure 1e), suggesting that CiBGLU21 may function in the cell membrane, cell wall, or extracellular region.

### 2.2. CiBGLU21 Promotes Graft Union Formation

**Figure 2:**
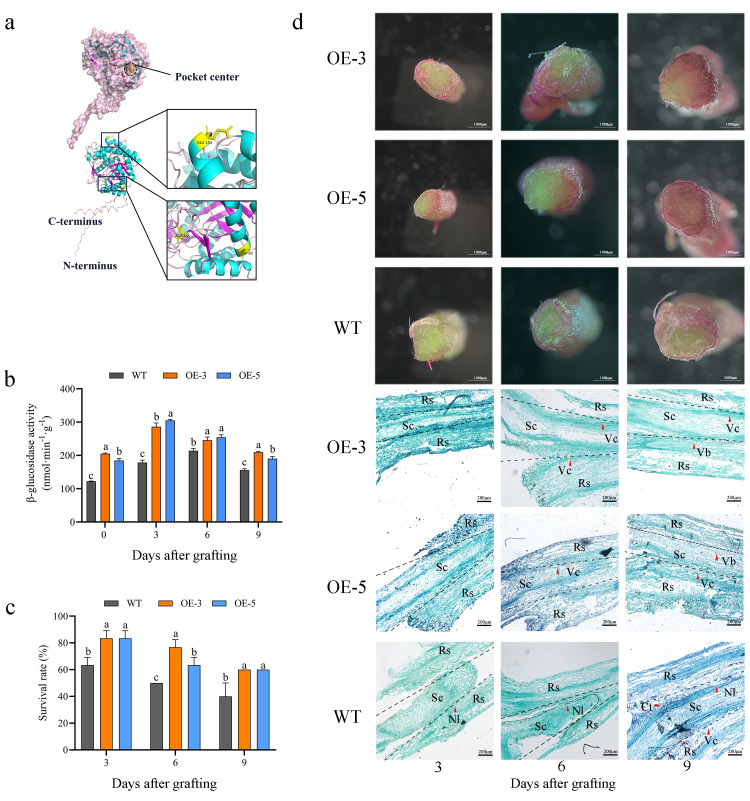
The functional analysis of *CiBGLU21* in graft healing. (**a**) A structural model of CiBGLU21, with three amino acids (Glu153, Asp465, and Asp92 marked in yellow) identified as catalytically active sites located at the center of the active pocket. (**b**) The β-glucosidase activity in graft junctions of *CiBGLU21*-OE and WT lines at 0, 3, 6, and 9 days after grafting. (**c**) The survival rate and (**d**) phenotypic comparison of graft unions in *CiBGLU21*-OE and WT plants at 3, 6, and 9 days after grafting. Sc: Scion, Rs: Rootstock, Nl: Necrotic Layer, Ct: Callus Tissue, Vb: Vascular Bundle, and Vc: Vascular Connection. Values represent mean ± SD; different letters represent a significant difference via Duncan’s multiple range test.

Using AlphaFold for protein structure predictions, CiBGLU21 was found to adopt a groove-like conformation, with a central region corresponding to the previously reported conserved active site motif of the BGLU family. This active site is formed by the residues Glu153, Asp465, and Asp92, which constitute a distinct catalytic pocket (Figure 2a). To validate the catalytic activity of CiBGLU21 across different developmental stages of the graft union, transgenic *CiBGLU21*-overexpressing (OE) poplar lines were generated (Supplementary Figure S3). The total β-glucosidase activity in both *CiBGLU21*-OE lines and wild-type (WT) plants showed an initial upregulation followed by downregulation. In contrast, the β-glucosidase activity was consistently elevated at all graft union stages in *CiBGLU21*-OE lines relative to the WT (Figure 2b).

To validate the functional role of *BGLU21* in graft union formation in *C. illinoinensis*, two transgenic lines with differing expression levels were selected and maintained under controlled tissue culture conditions for four weeks before grafting. *CiBGLU21*-OE transgenic plants exhibited a significantly higher survival rate after grafting compared to WT controls (Figure 2c). Subsequently, rootstocks were immersed in a 0.1% (*w*/*v*) acid fuchsin solution, and dye translocation was monitored in the scion tissue located 1 cm above the graft junction (Figure 2d). The enhanced accumulation of acid fuchsin was observed in grafting combinations involving *CiBGLU21*-OE transgenic plants. Meanwhile, anatomical studies were also performed to study the graft union formation of different grafting combinations (Figure 2d). Furthermore, a quantitative assessment of the relative acid fuchsin content confirmed that *CiBGLU21*-OE transgenic plants accumulated significantly higher levels of the dye (Supplementary Figure S4).

### 2.3. CiBGLU21 Promotes Lignin Biosynthesis and Sugar Accumulation in Graft Healing Process

**Figure 3:**
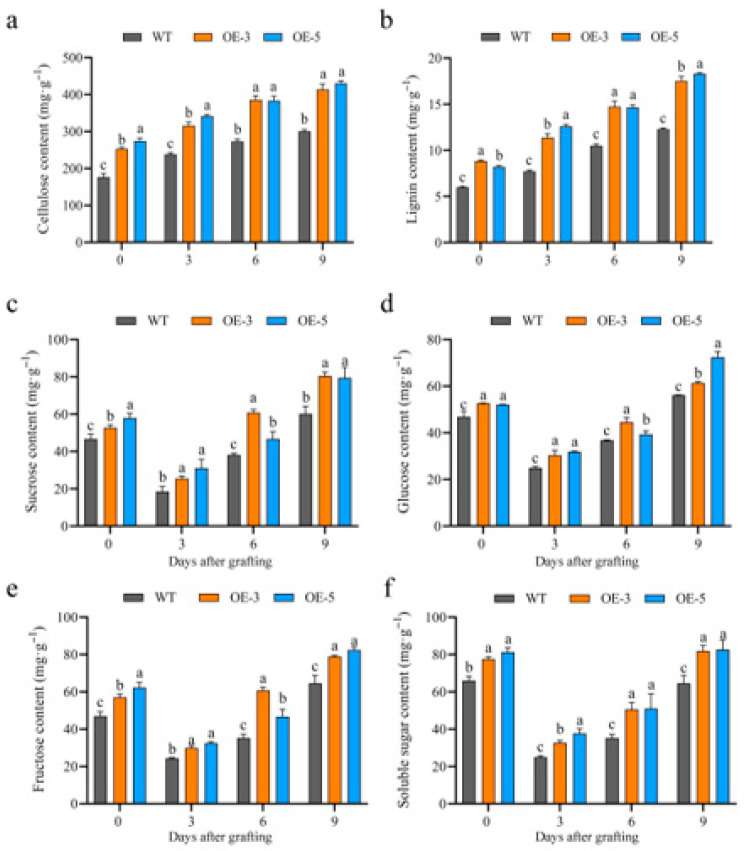
Comparison of total cellulose, lignin, sucrose, glucose, fructose, and soluble sugar contents between *CiBGLU21*-OE and WT plants (**a**–**f**). Values represent mean ± SD; different letters represent significant difference via Duncan’s multiple range test.

Cellulose and lignin were important substances for cell wall formation. Therefore, a comparative analysis of total cellulose and lignin contents revealed elevated levels of both components in the overexpressed transgenic lines relative to the WT throughout graft development (Figure 3a,b). Taken together, these data suggest that *CiBGLU21* positively regulated graft union formation by promoting the secondary metabolites of cell wall formation. Sugars are essential components that support rapid cell regeneration and cell wall formation.

To investigate the potential involvement of *CiBGLU21* in carbohydrate metabolism during graft union formation, the levels of sucrose, glucose, fructose, and soluble sugars—key energy sources and essential substrates for graft union development—were quantified in both *CiBGLU21*-OE and WT plants. The results showed that the levels of sucrose, glucose, fructose, and soluble sugars decreased following grafting and subsequently increased during graft union development (Figure 3a–d). Meanwhile, the contents of sucrose, glucose, fructose, and soluble sugars were higher in CiBGLU21-OE lines than in WT plants throughout the graft union formation process (Figure 3c–f).

### 2.4. CiWOX13 and CiWOX14 Directly Bind to the CiBGLU21 Promoter

**Figure 4:**
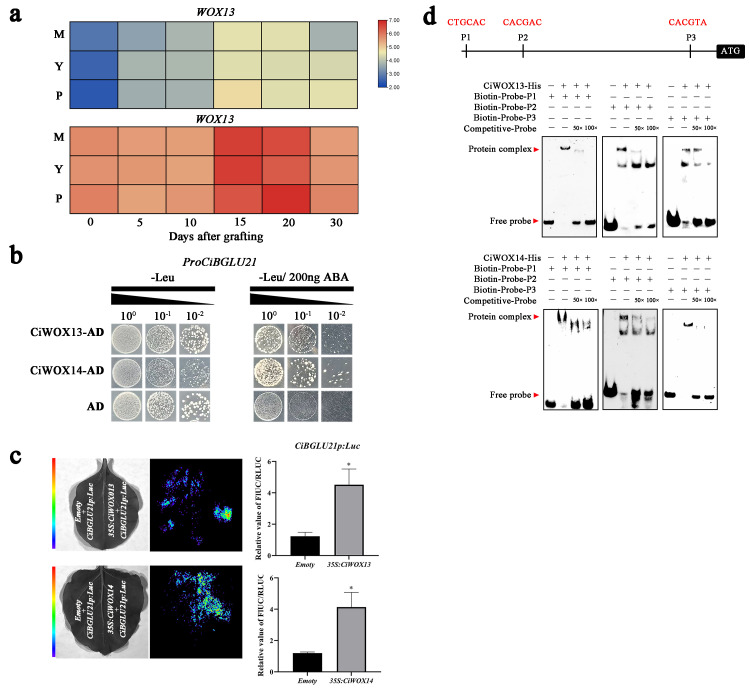
CiWOX13 and CiWOX14 directly bind to the *CiBGLU21* promoter. (**a**) The expression patterns of CiWOX13 and CiWOX14. M, the rootstock of the grafting combination is Mahan; P, the rootstock of the grafting combination is Pawnee; and Y, the rootstock of the grafting combination is Yalin. (**b**) The interaction of CiWOX13 and CiWOX14 with the *CiBGLU21* gene promoters verified by the Y1H analysis. (**c**) A transient LUC imaging assay demonstrates that CiWOX13 and CiWOX14 activate the transcription of the CiBGLU21-pro-LUC reporter gene. (**d**) The interaction between CiWOX13/14 with the *CiBGLU21* gene promoters in the EMSA. The G-boxes are highlighted in red. Error bars represent mean ± SD (n = 3). Statistical significance: * *p* < 0.01 (*t*-test).

The promoter sequence of *CiBGLU21* was cloned into the pAbAi vector to construct the pAbAi-CiBGLU21 reporter vector for yeast one-hybrid (Y1H) assays. A yeast nuclear protein library was constructed and screened using this promoter as bait to identify transcription factors that interact with it (Supplementary Table S4). Based on the expression patterns of candidate genes, we observed that CiWOX13 and CiWOX14 were upregulated during the grafting process (Figure 4a, Supplementary Table S5). Transformed yeast strains grew robustly on SD/-Leu/-Ura/AbA selective plates, whereas the negative control failed to grow (Figure 4b), confirming that CiWOX13 and CiWOX14 are capable of binding to the *CiBGLU21* promoter. This interaction was further validated by dual-luciferase (LUC) reporter assays (Figure 4c). To confirm the direct binding of CiWOX13 and CiWOX14 to the *CiBGLU21* promoter, an in vitro electrophoretic mobility shift assay (EMSA) was performed. Recombinant CiWOX13-His and CiWOX14-His proteins were heterologously expressed in *Escherichia coli* and purified. Biotin-labeled DNA probes derived from the *CiBGLU21* promoter region were incubated with the purified proteins. The results revealed the formation of specific DNA–protein complexes in reactions containing either CiWOX13-His or CiWOX14-His and the labeled probes encompassing G-box elements (Figure 4d). These findings demonstrate that CiWOX13 and CiWOX14 directly and specifically bind to the G-box motif within the *CiBGLU21* promoter. These results suggest that CiWOX13 and CiWOX14 interact with the G-box present in the promoters of *CiBGLU21*, thereby activating the expression of *CiBGLU21*.

### 2.5. The CiWOX13-CiWOX14 Complex Co-Promotes the Expression of CiBGLU21

**Figure 5:**
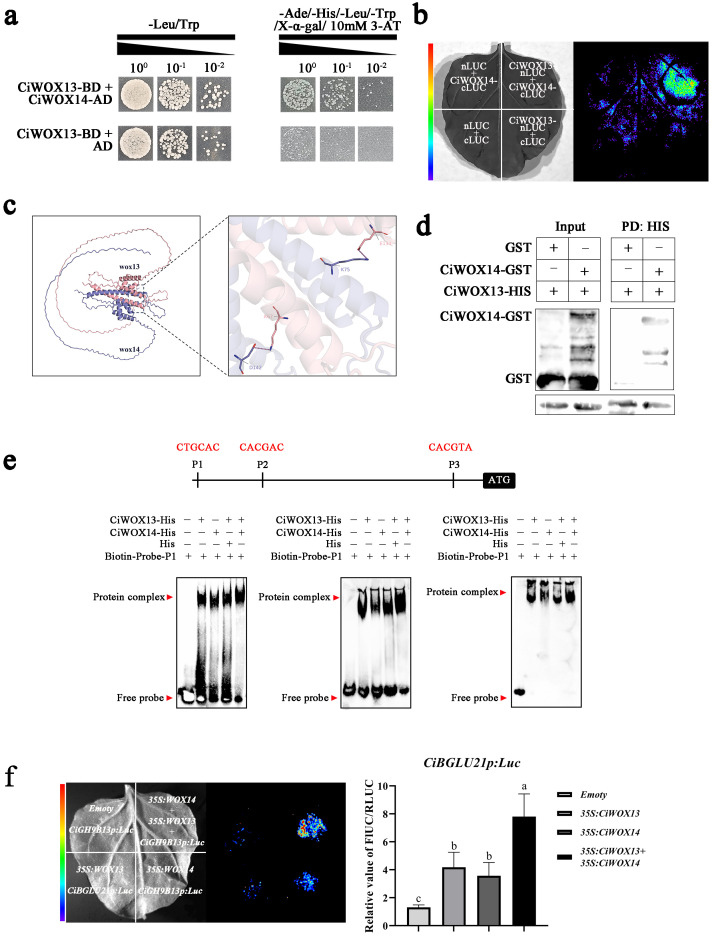
The CiWOX13-CiWOX14 complex plays a joint role in promoting the expression of *CiBGLU21*. (**a**) The Y2H assay indicates the interaction of CiWOX13 and CiWOX14 in yeast. (**b**) The analysis of the interaction between CiWOX13 and CiWOX14 in vivo using an LCI assay. The 35S-CiWOX13-nLuc and 35S-cLuc-CiWOX14 constructs were co-expressed in tobacco leaves. (**c**) The molecular docking between CiWOX13 and CiWOX14. (**d**) In vitro His pull-down assays between CiWOX13 and CiWOX14. (**e**) The interaction between CiWOX13/14 with the *CiBGLU21* gene promoters in the EMSA. The G-boxes are highlighted in red. (**f**) The transient LUC imaging assay demonstrates that CiWOX13 and CiWOX14, either alone or together, activate the transcription of the CiBGLU21-pro-LUC reporter gene. Values represent mean ± SD; different letters represent a significant difference via Duncan’s multiple range test.

To determine whether an interaction exists between CiWOX13 and CiWOX14, we conducted a yeast two-hybrid (Y2H) pairwise interaction assay. Yeast cells co-expressing pGBKT7-CiWOX13 and pGADT7-CiWOX14 grew normally on a selective medium, confirming the physical interaction between these two proteins (Figure 5a). Furthermore, firefly luciferase complementation imaging (LCI) and molecular docking analyses were employed to provide additional in vivo evidence for the interaction between CiWOX13 and CiWOX14 (Figure 5b,c). To delve deeper into this interaction, we purified CiWOX13 expressed in a prokaryotic system and performed pull-down experiments using GST and CiWOX14-GST (Figure 5d). The data established that CiWOX13 physically interacted with CiWOX14 and potentially acted in concert with the latter in the plant.

We performed an EMSA using CiWOX13 and CiWOX14 to examine the impact of the CiWOX13-CiWOX14 complex on the *CiBGLU21* promoter. In scenarios with an excess probe, when both CiWOX13 and CiWOX14 proteins are present at the binding site simultaneously, the shift band appears darker compared to when only one protein is present (Figure 5e). Subsequently, the fusion vector containing the 35S promoter was introduced into tobacco leaves. Specifically, empty 35S-CiWOX13 and 35S-CiWOX14 vectors were transformed into tobacco leaves. The findings indicated that the fluorescence intensity and enzyme activity were significantly higher when CiWOX13 and CiWOX14 were present in concert, compared to individual treatments (Figure 5f).

## 3. Discussion

Grafting technology has a long history in horticulture [33]. Graft healing is a complex development process that is affected by many factors, including the temperature and the genotype [34,35,36,37]. Graft union development is a process that involves cell division and differentiation at the graft junction [5,8,9]. Following the completion of cell regeneration, cellulose and lignin are important for cell walls formed in this process [1,38]. However, *BGLU* gene family studies are limited, and no reports exist regarding the *BGLU* gene in *C. illinoinensis*.

In the current investigation, we combined wet-lab experiments with a bioinformatic analysis to investigate the role of *BGLU* genes in the grafting union in *C. illinoinensis*. Utilizing the chromosome-level genome sequence, 22 *BGLU* genes were identified and named, reflecting their evolutionary relationships and gene IDs (Supplementary Table S3, Figure 1). The number of *BGLU* genes identified in pecan was less than that in *A. arabidopsis* [39], maize [40], and rice [41]. The phylogenetic analysis revealed six primary clusters within the *BGLU* gene family of *C. illinoinensis* (Figure 1a). The majority of members within the *CiBGLU* gene family possess a characteristic motif. Among the identified motifs, motifs 1, 2, 5, 6, 7, 8, 9, and 10 are widely conserved across most proteins, suggesting a high degree of evolutionary conservation. In contrast, motifs 3 and 4 are specific to certain members, and such unique motifs may represent distinct structural or functional features that could contribute to functional divergence; however, experimental validation is necessary to confirm their biological roles. Most members of the CiBGLU gene family possess both 5′- and 3′-UTRs, whereas these regions were detected in only two genes, namely CiBGLU7 and CiBGLU14 (Supplementary Figure S2b). Such structural variations in the gene architecture suggest a potential functional divergence among CiBGLU family members. The analysis of promoter sequences indicated that the elements were mainly concentrated in light-responsive, stress-responsive, growth–developmental, and hormone signaling elements (Supplementary Figure S2).

In pears, the *PbBglu13* gene encodes β-glucosidase and is more highly upregulated in the graft healing process, and β-glucosidase promotes grafting union [18]. In the current study, the expression patterns of the *CiBGLU* genes in the process of different grafting combinations are explored through a transcriptomic data analysis, and the activity of the β-glucosidase of different grafting combinations was tested, revealing that *CiBGLU21* is possibly important for graft union formation (Figure 1c,d). To validate our hypotheses and to understand the function of *CiBGLU21*, we cloned the *CiBGLU21* gene and confirmed its localization in the cell membrane (Figure 1e). The predictive results of the protein structure indicate that *BGLU21* contains a conserved active catalytic site motif of the *BGLU* family (Figure 2a). Compared to the WT, the β-glucosidase activities of *CiBGLU21*-OE were increased in the graft union process (Figure 2b). Furthermore, after grafting, *CiBGLU21*-OE transgenic lines displayed a higher survival rate, and the degree of graft union formation was significantly higher compared to WT (Figure 2d). Graft union is a progressive process of cell regeneration, and lignin and cellulose are important substances for cell biosynthesis and cell formation. In order to further study the function of *CiBGLU21* in the graft healing process, the lignin and cellulose contents of different grafting combinations of the WT and *CiBGLU21*-OE were tested. In our study, the lignin and cellulose contents were increased in *CiBGLU21*-OE transgenic lines (Figure 3a,b). Sugars serve as both respiratory substrates for energy generation and essential metabolic intermediates for macromolecule synthesis [17,18,42]. In addition, sugars also act as activators of important cell division and elongation processes [42]. Furthermore, sugar also had an important role in graft junction formation, and sugar responses, ranging from asymmetric to symmetric, were the main event in graft development [43]. In various plants, several studies have suggested that sucrose has a promoting effect for plant graft survival [43,44,45]. In this study, the accumulation of sugars (sucrose, glucose, fructose, and soluble sugar) was increased in the *CiBGLU21*-OE transgenic lines (Figure 3c–f). Thus, we propose that *CiBGLU21* promotes graft union by affecting the sugar and energy metabolism. These findings suggest that *CiBGLU21* plays an important positive role in promoting graft union by increasing substances for cell wall synthesis to promote graft healing. However, whether *CiBGLU21* was an important gene for enhancing the cell adhesion capacity of the rootstock and promoting graft union healing efficiency remains open for further experimental investigation.

Transcription factors (TFs) in plant cells modulate the transcription and expression of target genes by binding to specific cis-regulatory elements within their promoter regions, thereby regulating gene activity. Therefore, identifying upstream regulatory TFs is essential for elucidating the molecular mechanisms underlying graft union formation in *C. illinoinensis*. In this study, a 2000 bp promoter sequence located upstream of *CiBGLU21* was cloned and used to screen the Y1H library for interacting TFs (Supplementary Table S4). Two WOX family transcription factors were identified as potential regulators that may respond to wounding and contribute to graft union development through direct binding to the *CiBGLU21* promoter (Figure 4a). The WOX transcription factor family plays pivotal roles not only in regulating plant tissue and organ development but also in establishing and maintaining stem cell niches [46]. Multiple WOX members are well-documented regulators of stem cell formation and homeostasis [47, 48, 49]. Notably, CiWOX13 has been shown to specifically bind G-box-like motifs [9]. In this study, we demonstrated that both CiWOX13 and CiWOX14 directly interact with the *CiBGLU21* promoter, thereby positively regulating its expression (Figure 4b,c). In our study, we observed analogous binding specificity, as CiWOX13 and CiWOX14 demonstrated binding to the three G-box-like motifs within the *CiBGLU21* promoter (Figure 4d). In transcription factor regulation, the interaction between multiple transcription factors plays a positive role in regulating changes in downstream genes; we also observed an interaction between CiWOX13 and CiWOX14 (Figure 5a–d). The EMSA results showed that the presence of both CiWOX13 and CiWOX14 significantly enhanced the DNA–protein complex formation at the G-box element (Figure 5e). Furthermore, the co-expression of CiWOX13 and CiWOX14 led to a significant increase in the LUC reporter expression compared to the expression driven by either protein alone (Figure 5f).

**Figure 6:**
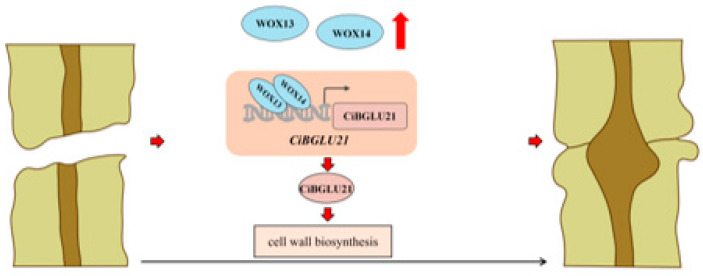
The proposed model of *CiBGLU21* functions in graft union formation in *C. illinoinensis*. *CiBGLU21* directly and positively facilitates graft union formation by promoting activity and cell wall biosynthesis through the direct and synergistic binding of transcription factors CiWOX13 and CiWOX14 to the promoter of *CiBGLU21*.

Finally, we established a model proposing the role of *CiBGLU21* in graft union formation (Figure 6). In this model, *CiBGLU21* facilitates graft union formation by regulating cell wall biosynthesis and sugar metabolism. Additionally, CiWOX13 and CiWOX14 directly bind to the G-box element in the promoter of *CiBGLU21* to positively regulate its expression. Moreover, the CiWOX13–CiWOX14 complex synergistically activates *CiBGLU21* by binding to its promoter. This study offers insights into the molecular mechanisms underlying *CiBGLU21*-mediated graft union formation in woody plants.

## 4. Materials and Methods

### 4.1. Plant Materials and Grafting

**Figure 7:**
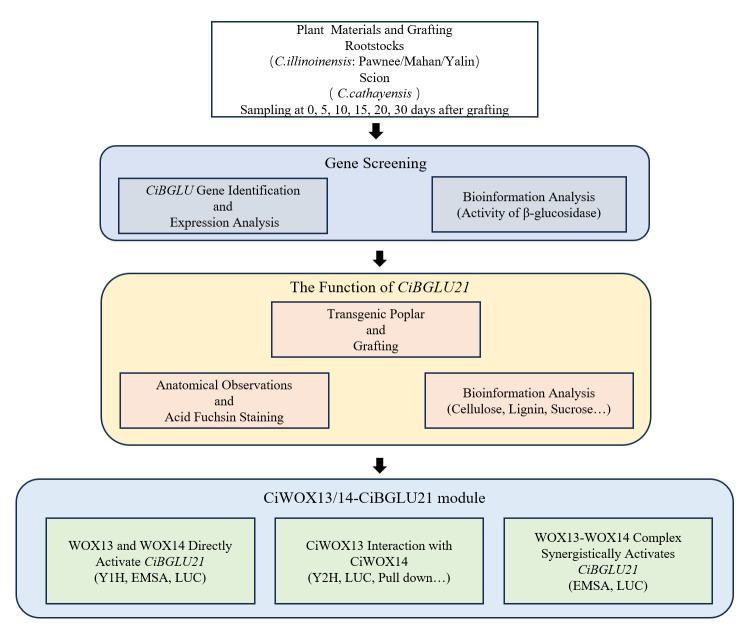
A schematic overview of the experimental design.

One-year-old Chinese hickory cuttings were used as scions, and one-year-old seedlings of three pecan cultivars (“Mahan”, “Pawnee”, and “Yalin”) were used as rootstocks for cleft grafting. The seedlings were planted in pots with a diameter of 17 cm and a height of 22 cm and cultivated in the Regional Experimental Park of Zhejiang A&F University. Graft union tissues were collected at 0, 5, 10, 15, 20, and 30 days after grafting. Three biological replicates were conducted at each time point. Collected samples were immediately frozen in liquid nitrogen and subsequently stored at −80 °C. The experimental workflow is illustrated in the following figure (Figure 7).

### 4.2. The Identification and Physicochemical Properties of the BGLU Proteins in C. illinoinensis

Candidate gene sequences of *BGLU* in *C. illinoinensis* were screened from the published genome *C. illinoinensis* Pawnee_v1 (https://www.ncbi.nlm.nih.gov/, accessed on 14 November 2023). A local BLASTp search was conducted using *A. thaliana* BGLU proteins as a seed file (built-in TBtools v2.097). HMMER 3.3 software was used to screen for candidate *BGLU* gene family members in *C. illinoinensis,* through which the conserved domain of BGLU was retrieved from the Pfam database. The results from BLAST and HMMER were integrated to eliminate incomplete and redundant protein sequences. The online software ExPASy (https://web.expasy.org/protparam/ accessed on 19 December 2024) was used to perform protein analysis. Cell-PLoc2.0 (http://www.csbio.sjtu.edu.cn/bioinf/Cell-PLoc-2/ accessed on 19 December 2024) was used to predict the subcellular localizations of CiBGLU proteins.

### 4.3. Phylogenetic, Gene Structure, Collinearity, Chromosomal Localization, and Prediction Analyses of Cis-Elements in the Promoters of CiBGLUs

The protein sequences of BGLU from *C. illinoinensis*, *A. thaliana*, *O. sativa*, *Z. mays*, and *P. trichocarpa* (Supplementary Table S1) were retrieved and aligned using MEGA 7.0 to construct a neighbor-joining phylogenetic tree with 2000 bootstrap replicates. The resulting tree was visualized using the online software iTOL (https://itol.embl.de/ accessed on 19 December 2024).

The NCBI Conserved Domain Database (CDD) (https://www.ncbi.nlm.nih.gov/Structure/cdd/wrpsb.cgi accessed on 19 December 2024) was used to confirm the presence of conserved domains in BGLU proteins. Gene structure analysis was performed based on annotations from the reference genomes. The MEME suite (http://meme-suite.org/ accessed on 20 December 2024) was employed to identify conserved motifs within BGLU proteins of *C. illinoinensis*, with the maximum number of motifs set to ten; the identified motifs were subsequently downloaded. Phylogenetic relationships, conserved motif architectures, and gene structure comparisons were integrated and visualized using TBtools v2.097.

Collinearity analyses among *BGLU* gene families in *C. illinoinensis*, *A. thaliana*, and *P. trichocarpa* were conducted using TBtools v2.097. Chromosomal localization of BGLU genes in *C. illinoinensis* was determined by extracting genomic position information from genome and annotation files, and a physical map of these genes on chromosomes was generated using TBtools v2.097.

To identify putative cis-acting regulatory elements in the promoter regions, the 2000 bp upstream sequences of all *BGLU* genes were analyzed using the PLANTCARE database (https://bioinformatics.psb.ugent.be/webtools/plantcare/html/ accessed on 20 December 2024), and the results were visualized with TBtools v2.097.

Protein structure prediction and molecular docking were performed using the AlphaFold3 web server (https://alphafoldserver.com/welcome accessed on 21 August 2025).

### 4.4. Library Construction and Sequencing

A total of 1 µg of RNA per sample was prepared for analysis. A library was constructed using the chain-specific construction method, and the insert size of the library was assessed using an Agilent 2100 bioanalyzer to determine the library quality. Sequencing was performed on an Illumina Novaseq™ 6000 (https://www.lc-bio.com, LC-Bio Technology CO., Ltd., Hangzhou, China), following the vendor’s recommended protocol. Fastp software v0.10.1 was used to remove the reads that contained adaptor contamination, low-quality bases, and undetermined bases with default parameters. Then, sequence quality was also verified using fastp. We used HISAT2 to map reads to the reference genome of *C. illinoinensis* Pawnee_v1. Gene annotation files were downloaded from the NCBI website (https://www.ncbi.nlm.nih.gov/ accessed on 14 November 2023). The mapped reads of each sample were assembled using StringTie with default parameters. StringTie was used to assess expression level for mRNAs by calculating FPKM.

### 4.5. RT-qPCR Analysis

RNA was extracted using FastPure Plant Total RNA Isolation Kit (RC401, Vazyme, Nanjing, China), and cDNA was synthesized by reverse transcription (R211, Vazyme, Nanjing, China). RT-qPCR analysis was conducted using ChamQ SYBR Color qPCR Master Mix (Q411, Vazyme, Nanjing, China). All primers are listed in Supplementary Table S2.

### 4.6. Plasmid Construction, Subcellular Localization, and Genetic Transformation

The coding sequence of *CiBGLU21* was cloned from the cDNA library of *C. illinoinensis* and recombined into the pCAMBIA1300-GFP vector. The recombinant 35S-CiBGLU21-GFP vector was used to transform *Agrobacterium tumefaciens* GV3101. *A. tumefaciens* GV3101 harboring 35S-CiBGLU21-GFP constructs was used for subcellular localization and genetic transformation.

Subcellular localization was used for transient transformation of 45-day-old tobacco leaves and was performed as previously described in Yang et al. [28]. After culturing for 48 h, the fluorescence signal in the transformed tobacco leaves was detected using a confocal microscope (LSM880, Zeiss, Oberkochen, Germany) with 488 and 594 nm argon lasers.

The 35S-CiBGLU21-GFP construct was introduced into “84K” (*Populus alba* × *P. glandulosa*) plants by *A. tumefaciens* strain GV3101-mediated transformation according to the protocol described by Tsuyama and Takabe [24]. The transgenic plants were identified by sequencing and examining transgene expression. Both WT and transgenic “84K” tissue culture plants were grown on WPM medium with 1.0 mg·L^−1^ 6-BA, 0.3 mg·L^−1^ IBA, and 30 g·L^−1^ sucrose and 7 g·L^−1^ agars in a tissue culture room. The uniform tissue plants of WT and transgenic “84K” that were about 5 cm in length were selected and used as rootstocks and scions to compose different grafting combinations by splice grafting.

### 4.7. Anatomical Observations and the Acid Fuchsin Staining Method

The samples of the grafting junction were collected at different stages during the graft healing process in different “84K” grafting combinations for anatomical and histological observations. Samples were sectioned to 10 μm vertically using a rotary microtome (RM2235, Leica, Wetzlar, Germany), dewaxed, rehydrated, cleaned, stained with toluidine blue, counterstained with safranin, and then fixed with neutral balata. Sections were examined with a light microscope (Axio Imager 2, Zeiss, Oberkochen, Germany), and representative sections were photographed.

Alternatively, the acid fuchsin staining method was used for quantitative determination of xylem transport capacity. The relative contents of acid fuchsin were assessed as previously described in Cui et al. [24].

### 4.8. Construction and Screening of Yeast Library

The cDNA nuclear library from *C. illinoinensis* leaves was constructed through RNA extraction, mRNA purification, reverse transcription, amplification, and bacterial transformation. Additionally, the *CiBGLU21* promoter was cloned into the pABAi vector to screen for interaction proteins, as detailed in Supplementary Table S4.

### 4.9. Yeast One-Hybrid Analysis, Dual-Luciferase Reporter Assay, and Electrophoretic Mobility Shift Assay

The *CiBGLU21* promoter sequence was cloned based on genome sequencing data and inserted into the pAbAi vector, followed by transformation into the Y1H Gold yeast strain. An empty pGADT7 vector was introduced into the recombinant yeast strain as a control and plated onto SD/-Leu/-Ura plates supplemented with AbA to determine the appropriate AbA concentration. The sequences of the target genes (CiWOX13 and CiWOX14) were cloned into the expression vector pGADT7 and subsequently transformed into the recombinant yeast strains. Individual clones were selected and incubated on SD/-Leu/-Ura plates containing 200 ng/mL AbA for 3 days at 30 °C.

The promoter sequence of *CiBGLU21* was cloned into the pGreen II-0800-Luc vector and co-transformed with the pGreen II-62-SK vector containing the CiWOX13 and CiWOX14 gene sequences into *A. tumefaciens* strain GV3101 (pSoup-p19). Transient transformation of tobacco was performed using the *A. tumefaciens*-mediated expression system, followed by incubation in darkness for 24 h and subsequent exposure to normal light for 2 d. Prior to imaging, plants were kept in the dark for 15 min, and LCI images were captured using a low-light cooled charge-coupled device camera (Tanon 5200 Multi, Shanghai, China).

The recombinant proteins CiWOX13-His and CiWOX14-His were purified and subsequently incubated with biotin-labeled DNA fragments, while unlabeled DNA fragments were used as cold probes. EMSA analysis was conducted using the Chemiluminescent EMSA Kit (MH104, Coolaber, Beijing, China) according to the manufacturer’s instructions. Primer sequences are provided in Supplementary Table S2.

### 4.10. Yeast Two-Hybrid Analysis, Firefly Luciferase Complementation Assay, and Pull-Down Assay

The CDSs of CiWOX13 and CiWOX14 were cloned into the pGADT7 and pGBKT7 vectors, respectively, resulting in fusion proteins containing the activation domain (AD) and the DNA-binding domain (BD). The AD-CiWOX13 and BD-CiWOX14 plasmids were transformed into yeast cells using the Yeast Two-Hybrid Interaction Proving Kit (YM2001, Coolaber, Beijing, China) according to the manufacturer’s instructions.

In the His pull-down assay, the full-length cDNA of CiWOX13 was cloned into the pET28a vector, while the full-length cDNA of CiWOX14 was inserted into the pGEX-6P-1 vector. The His pull-down assay was performed with the His pull-down kit (PC4410, Solarbio, Beijing, China). The interaction between CiWOX13 and CiWOX14 was analyzed by SDS-PAGE and Western blotting.

The CDSs of CiWOX13 and CiWOX14 were separately inserted into the pCAMBIA1300-nLuc and pCAMBIA1300-cLuc vectors to generate LUC reporter constructs. *A. tumefaciens* cultures harboring the respective plasmids were co-infiltrated into tobacco leaves, followed by incubation in darkness for 24 h and subsequent exposure to normal light for 2 d. Prior to imaging, plants were kept in the dark for 15 min, and LCI images were captured using a low-light cooled charge-coupled device camera (Tanon 5200 Multi, Shanghai, China). Primer sequences are provided in Supplementary Table S2.

### 4.11. Statistical Analysis

One-way analysis of variance was used to analyze differences among the experimental groups. IBM SPSS 19.0 statistical software (IBM, Armonk, NY, USA) and Microsoft Excel 2010 were used for statistical analysis, and GraphPad Prism 8.0 (GraphPad Software, La Jolla, CA, USA) was used for visualization.

## 5. Conclusions

In this study, we identified *CiBGLU21*, an important β-glucosidase gene in pecans (*C. illinoinensis*), as an important regulator of graft union formation. Through a genome-wide analysis, we demonstrated that *CiBGLU21* promoted graft healing by enhancing β-glucosidase activity, which drives the lignin and cellulose deposition essential for cell wall reinforcement. We further discovered that the transcription factors CiWOX13 and CiWOX14 directly and synergistically activate *CiBGLU21* by binding to its promoter, thereby linking wound-induced signaling to cell wall remodeling. These findings provide the first mechanistic insights into grafting success in pecans, highlighting the CiWOX13/14-*CiBGLU21* module as a central regulatory axis. The results offer a foundation for developing strategies to enhance grafting efficiency in pecans and other crops, bridging fundamental research with agricultural innovation. Future studies should investigate whether this regulatory pathway is conserved in other woody species, with potential implications for improving perennial crop performance more broadly.

## Data Availability

The original contributions presented in this study are included in the article/Supplementary Materials. Further inquiries can be directed to the corresponding authors.

## Supplementary Materials

The following supporting information can be downloaded at https://www.mdpi.com/article/10.3390/plants15020273/s1: Figure S1: Phylogenetic Analysis, Gene Structure, and Motif Analysis of CiBGLU Genes; Figure S2: The collinearity analysis, chromosomal location, and promoter analysis of CiBGLU genes; Figure S3: Identification of transgenic populus plants; Figure S4: Total contents of acid fuchsin between the wild-type and the transgenic lines; Table S1: Information about genes from other species used in phylogenetic analysis; Table S2: Primers used in this study.; Table S3: Information of CiBGLU genes and properties of the deduced proteins; Table S4: The results of Y1H; and Table S5: Information on CiWOXs TFs.
