# Supplementary material for: The CiWOX13-CiWOX14 Complex Regulates CiBGLU21 to Promote Graft Union Formation by Modulating Cell Wall Synthesis in Carya illinoinensis
Source: Plants (Basel). 2026 Jan 16;15(2):273. doi: 10.3390/plants15020273 (PMC12844663; doi:10.3390/plants15020273)
Supplement: Supplementary file 1 [file plants-15-00273-s001.zip › plants-4045921-Figures.pdf]

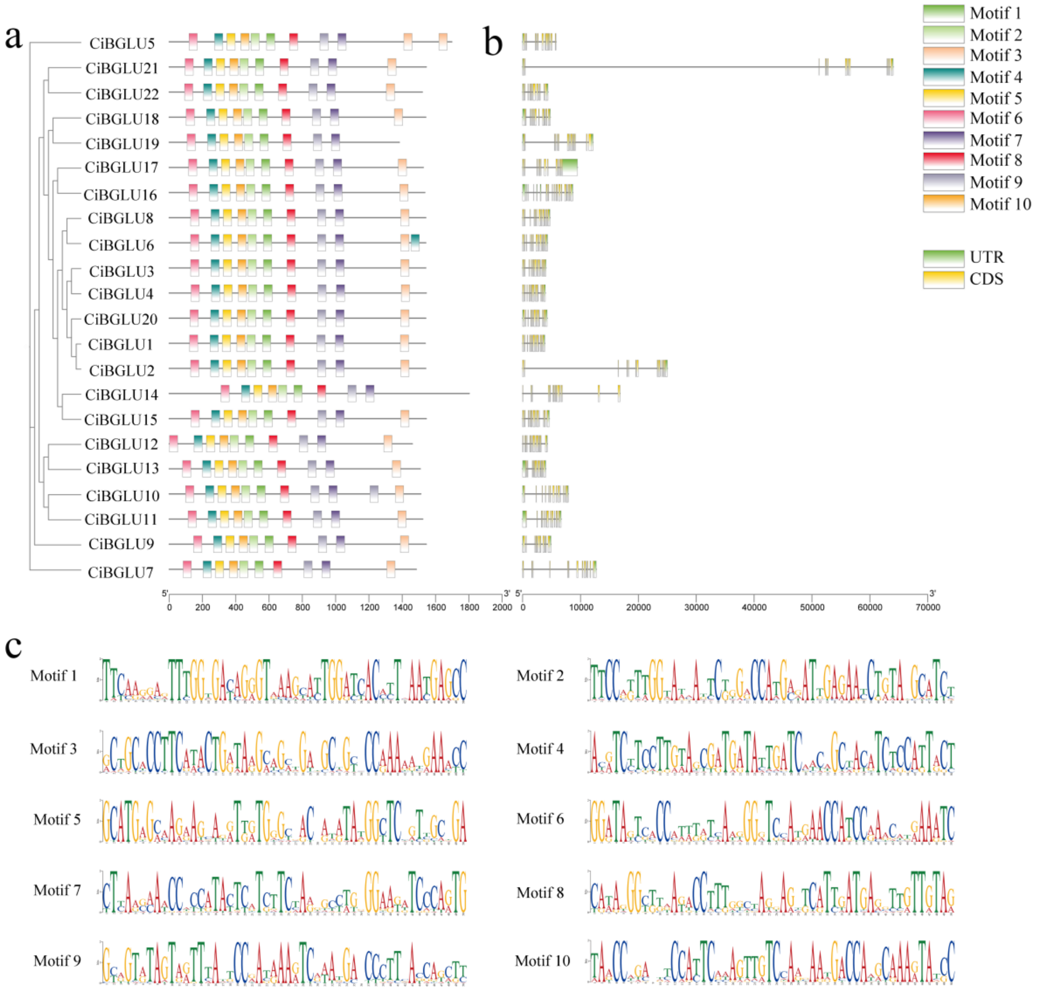

Supporting Figure S1. Phylogenetic analysis, gene structure, and motif analysis of *CiBGLU* genes. (a) Phylogenetic relationships and motif distribution. (b) Analysis of *CiBGLU* family gene structure. (c) Motif information.

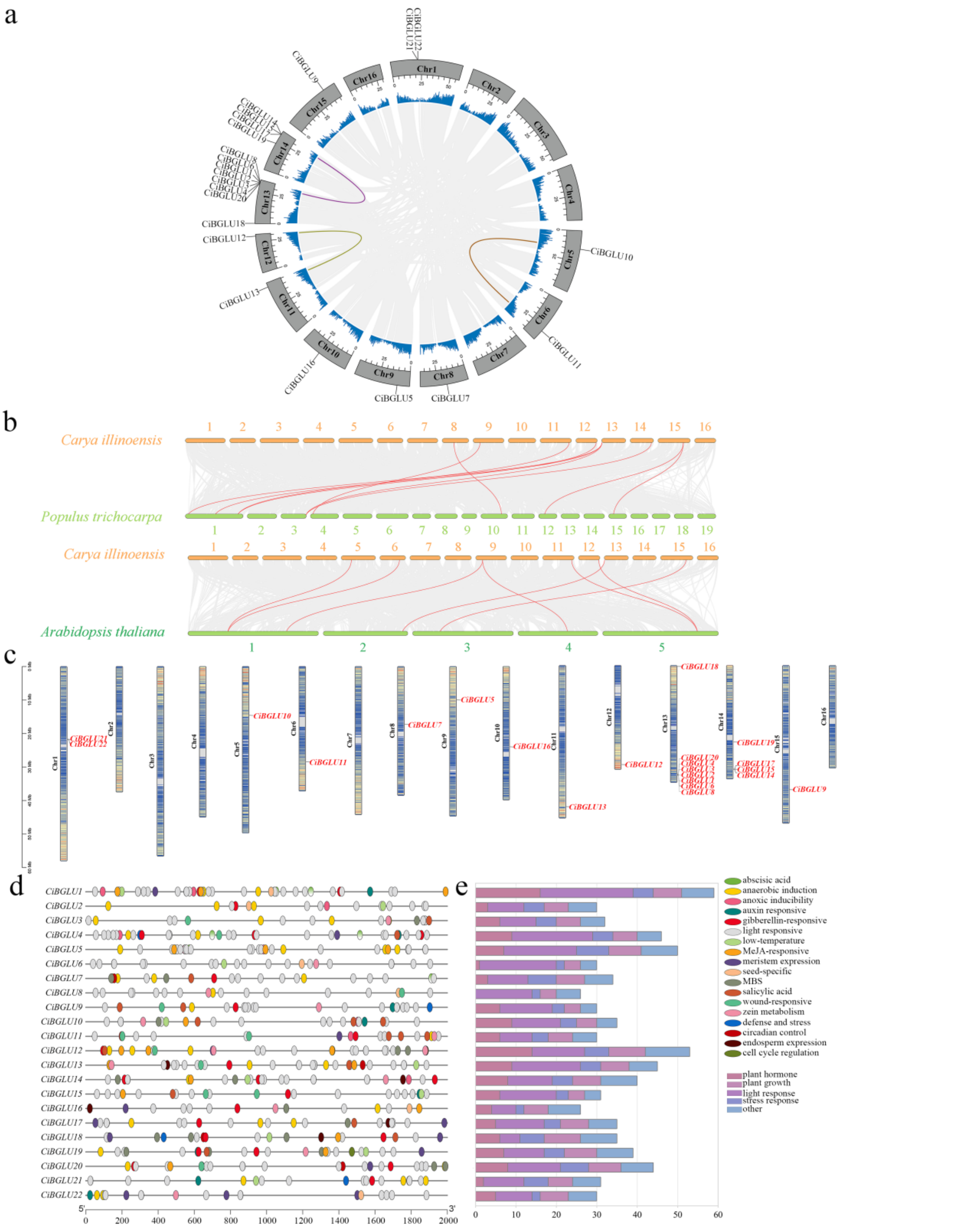

Supporting Figure S2. The collinearity analysis, chromosomal location, and promoter analysis of *CiBGLU* genes. (a) Synteny analysis of *C. illinoinensis*. The lines represent segmental duplicated gene pairs. (b) Collinear analysis of BGLU proteins in *C. illinoinensis* and other plants. The red lines indicate the chromosome collinearity of BGLU proteins in different species. The gray line represents the synteny blocks between *C. illinoinensis*, *P. trichocarpa,* and *A. thaliana.* (c) Chromosomal location. (d) The distribution of cis-acting elements at 2000 bp upstream, and (e) the number of

light response, plant hormone, plant growth, and stress response elements for each *CiBGLU* gene.

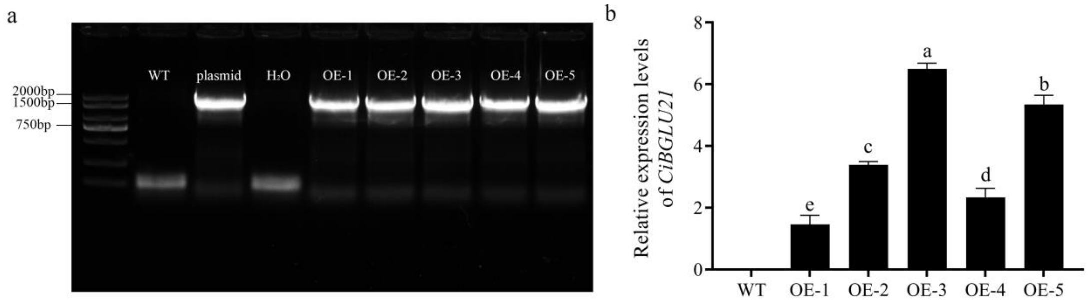

Supporting Figure S3. Identification of transgenic populus plants. (a) The PCR analysis of the transgenic populus by gene-specific primers. (b) The expression analysis of *CiBGLU* in the transgenic populus and wild type.

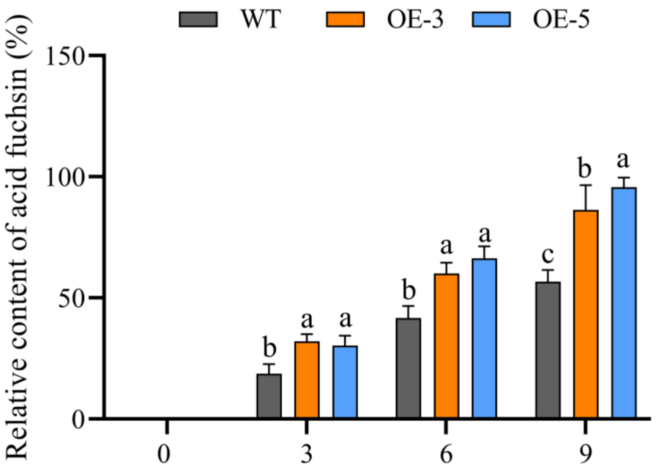

Supporting Figure S4. Total contents of acid fuchsin between the wild-type and the transgenic lines (a-f). Values represent mean±SD; different letters represent significant difference via Duncan's multiple range test.
